# Preoperative predictive thresholds for successful canaloplasty in glaucoma patients

**DOI:** 10.1007/s00417-025-06887-6

**Published:** 2025-07-04

**Authors:** Julia Prinz, Carla-Maria Epping, David Kuerten, Matthias Fuest, Claus Cursiefen, Peter Walter, Karl Mercieca, Verena Prokosch

**Affiliations:** 1 Department of Ophthalmology, Faculty of Medicine, University Hospital of Cologne, 50937 Cologne, Germany; 2 Department of Ophthalmology, RWTH Aachen University, 52074 Aachen, Germany; 3 Department of Ophthalmology, University Hospital of Bonn, 53127 Bonn, Germany

**Keywords:** Glaucoma, Canaloplasty, Intraocular pressure, Logistic regression

## Abstract

**Purpose:**

To establish preoperative thresholds of intraocular pressure (IOP) levels and the number of IOP-lowering eye drops that predict the surgical success of canaloplasty.

**Methods:**

This retrospective study included 166 glaucoma patients undergoing canaloplasty. Data on age, sex, glaucoma subtype, visual acuity, IOP, number of IOP-lowering eye drops, intraoperative and postoperative complications, and reoperations were collected during a 12-month follow-up period. Logistic regression models were applied to identify preoperative thresholds associated with an increased risk of surgical failure.

**Results:**

At 12 months, mean IOP decreased from 24.2 ± 7.8 to 14.8 ± 3.7 mmHg, and the number of IOP-lowering eye drops from 2.3 ± 1.1 to 0.6 ± 1.0 (both *p* < 0.001). For success rates ≤ 18 and ≤ 15 mmHg, logistic regression identified a preoperative IOP threshold of 36.9 and 27.1 mmHg (qualified success), and 27.1 and 20.1 mmHg (complete success), respectively, above which the likelihood of surgical failure increased. No significant association was found between the number of preoperative IOP-lowering eye drops and surgical success. No long-term postoperative complications were recorded.

**Conclusions:**

In patients with preoperative IOPs of ≤ 36.9 mmHg or 27.1 mmHg, canaloplasty is likely to be an effective and safe procedure to obtain target pressures ≤ 18 mmHg with or without IOP-lowering eye drops, respectively. These data suggest that preoperative thresholds could help predict postoperative outcomes and improve patient selection for glaucoma surgery.

## Key messages

*****What is known***:**

- Canaloplasty is an effective surgical technique for lowering intraocular pressure (IOP) in glaucoma patients, but precise preoperative factors influencing its success remain unclear.

*****What is new***:**

- In our study, we identified IOP thresholds (≤ 36.9 mmHg for qualified success and ≤ 27.1 mmHg for complete success) that may predict the likelihood of achieving target pressures ≤ 18 mmHg with or without IOP-lowering eye drops, respectively.
- In our study, the number of preoperative IOP-lowering eye drops did not significantly impact the success of canaloplasty, suggesting that baseline IOP is a more critical predictor.
- These findings provide valuable preoperative criteria that can enhance patient selection, potentially improving surgical outcomes for glaucoma patients undergoing canaloplasty.

## Introduction

Glaucoma is a group of diseases characterized by progressive optic nerve damage, commonly associated with elevated intraocular pressure (IOP) [1, 2]. Worldwide, glaucoma is a leading cause of blindness, highlighting the importance of enhancing medical and surgical interventions to preserve vision [3, 4]. Traditional treatment modalities include pharmacological therapy, laser treatments, and various surgical techniques designed to lower IOP [5]. Among these techniques, canaloplasty is a well-established non-penetrating surgical method that aims at enhancing the physiological outflow of aqueous humour through Schlemm’s canal [6]. Unlike traditional filtering surgeries such as trabeculectomy, canaloplasty avoids the formation of a filtering bleb, thus minimizing complications such as hypotony or bleb-related infections [7, 8]. Several studies have demonstrated that canaloplasty effectively lowers IOP and reduces the dependence on IOP-lowering eye drops [9, 10].

However, the success of canaloplasty appears to be influenced by various preoperative factors [10]. Elevated baseline IOP and the number of preoperative IOP-lowering eye drops have been suggested to affect outcomes of canaloplasty [10]. Several studies have shown that patients with extremely high IOP levels may be less likely to benefit from canaloplasty [9]. To date, precise preoperative IOP criteria for surgical success are poorly defined. Identifying such thresholds could improve surgical outcomes by optimizing patient selection and tailoring the surgical approach to individual patient profiles.

The aim of the present two-centre study, involving 166 patients undergoing canaloplasty, is to determine critical preoperative thresholds—specifically, the maximum acceptable IOP and number of IOP-lowering eye drops—that may predict a successful outcome following canaloplasty.

## Materials and methods

### Patient selection

In this retrospective study, we included a consecutive series of 166 eyes of 166 patients undergoing canaloplasty. All patients were treated at the Department of Ophthalmology at the University Hospital of Cologne or Department of Ophthalmology, RWTH Aachen University Hospital. The study adhered to the tenets of Helsinki. It was approved by the medical ethics committee of the University of Cologne (21-1286_2) and RWTH Aachen University (EK 015/15, 410/20).

The baseline characteristics of all included patients are detailed in Table 1. All patients underwent clinical examinations preoperatively and at 1 day, 1 month, 6, and 12 months after canaloplasty. The examinations included measurement of IOP, the number of IOP-lowering eye drops, and the best corrected visual acuity (BCVA), measured in logarithm of the minimum angle of resolution (logMAR). Additionally, the visual field mean deviation (MD) values of performed static computer perimetry using the Humphrey Field Analyzer (Model 750, Humphrey-Zeiss, San Leandro, CA, USA), employing the white-on-white 24 − 2 full threshold (SITA program) were recorded. Intra- and postoperative surgery-related complications were also evaluated.

**Table 1:** Patient characteristics at baseline

Characteristics	*n*	%
n	166	
Age [years]	65.8 ± 13.1	
Female	87	52.4%
MD [dB]	−8.9 ± 8.1	
IOP [mmHg]	24.2 ± 7.8	
IOP-lowering eye drops (mean)	2.3 ± 1.1	
Glaucoma subtype		
POAG	129	77.7%
PSXG	23	13.9%
Pigmentary Glaucoma	9	5.4%
NTG	5	3.0%

*MD* mean deviation of visual field testing, *IOP* intraocular pressure, *POAG* primary open angle glaucoma, *PSXG* pseudoexfoliative glaucoma, *NTG* normal tension glaucoma

Only patients with a MD of no worse than − 12 dB were included, ensuring that only moderate glaucoma cases were considered, while advanced stages were excluded. A minimum follow-up period of six months was required for inclusion. Patients with a prior history of glaucoma surgery were excluded from the study as were patients with a diagnosis of angle-closure glaucoma or neovascular glaucoma.

The surgery was considered a complete success if the IOP was lower than 21, 18, 15, or 12 mmHg, without IOP-lowering medications, whereas qualified success was defined as IOP lower than 21, 18, 15, or 12 mmHg, respectively, with IOP-lowering medications.

### Surgical technique

The technique of canaloplasty has been described previously [10]. Initially, a conjunctival flap was created at the 12 o’clock limbal position. Subsequently, a superficial scleral flap measuring 4 × 4 mm was created, and a second, deeper scleral flap of 1.5 × 3 mm was dissected, extending into the clear cornea while leaving only a few layers of scleral tissue intact. The roof of the Schlemm’s canal was detached and the deep scleral flap was removed.

A flexible, illuminated microcatheter (iTrackTM250A, iScience Interventional Corporation, Menlo Park, USA) was inserted into Schlemm’s canal and advanced 360 degrees. Every 2 clock hours, 1.4% sodium hyaluronate (HealonGV, Advanced Medical Optics Inc., Santa Ana, USA) was injected into the canal, guided by the blinking light at the microcatheter’s tip.

Finally, a 10 − 0 non-absorbable polypropylene suture (Prolene, Ethicon, Johnson & Johnson Medical Corporation, New Brunswick, USA) was tied to the microcatheter tip and threaded through Schlemm’s canal as the catheter was withdrawn, after which the suture was tightened to apply moderate tension to the inner wall of the canal. The superficial flap was closed with 10 − 0 non-absorbable nylon sutures (Ethicon), followed by conjunctival closure using 8 − 0 Vicryl sutures (Ethicon).

### Statistical analysis

Statistical analysis was performed using the Statistical Package for Social Sciences (IBM SPSS Statistics for Windows, Version 25, Armonk, NY: IBM Corp.) and GraphPad Prism (GraphPad Software Inc.; San Diego, CA, USA, Version 9.0 for Windows). All values are displayed as mean ± standard deviations (SD).

Between-group comparisons for continuous data were performed using one-way repeated measures analysis of variance (ANOVA). Categorical variables were analysed using Chi-square tests and Fisher’s exact tests. Kaplan-Meier survival curves were generated to evaluate the time to surgical failure, based on our definitions of surgical success (IOP ≤ 21, 18, 15, or 12 mmHg). Logistic regression models were employed to determine critical preoperative thresholds of IOP and the number of IOP-lowering eye drops which were predictive of surgical failure. A p-value of ≤ 0.05 was considered statistically significant for all analyses, and confidence intervals (CI) were reported where applicable.

## Results

**Fig. 1:**
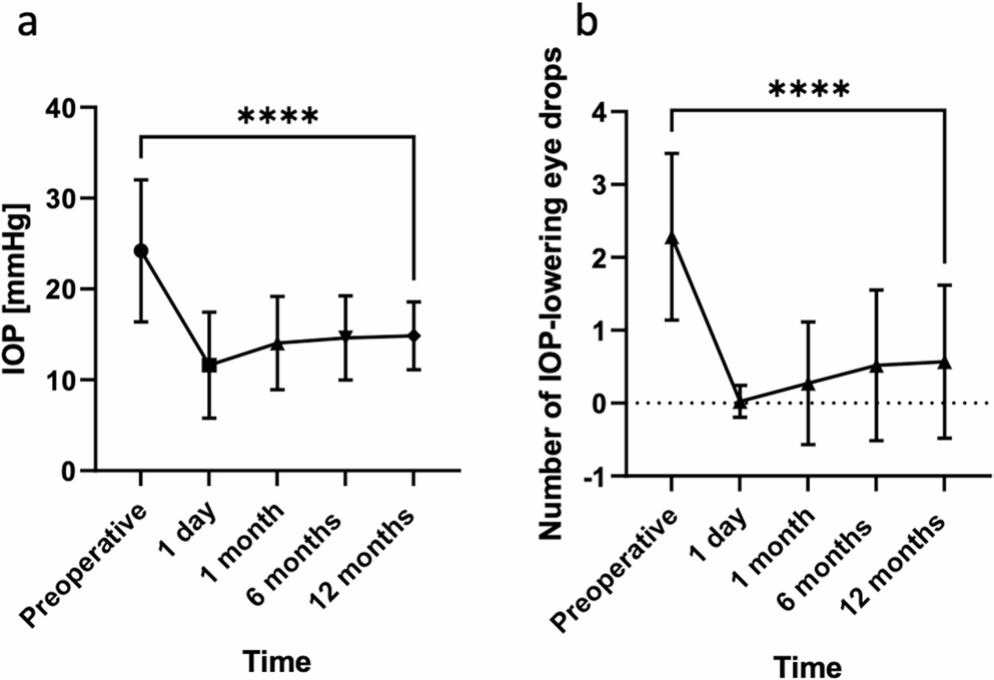
Mean intraocular pressure (IOP, mmHg, **a**) and number of IOP-lowering eye drops (**b**) preoperatively and 1 month, 3, 6, and 12 months following canaloplasty. **** *p* < 0.001

The baseline mean IOP was 24.2 ± 7.8 mmHg and was significantly reduced to 11.6 ± 5.8 mmHg at the first postoperative day (*p* < 0.001). At the 1-, 6- and 12-month follow-ups, the IOP was reduced significantly to 14.1 ± 5.1, 14.6 ± 4.6, and 14.8 ± 3.7 mmHg, respectively (*p* < 0.001, Fig. 1a). The relative IOP reduction was 34.0%.

The mean preoperative number of IOP-lowering eye drops was 2.3 ± 1.1. During the follow-up period, it was significantly reduced to 0.0 ± 0.2 at the first postoperative day, 0.3 ± 0.8 at the 1-month follow-up, 0.5 ± 1.0 at the 6-month follow-up, and 0.6 ± 1.0 at the 12-month follow-up (*p* < 0.001, Fig. 1b).

**Fig. 2:**
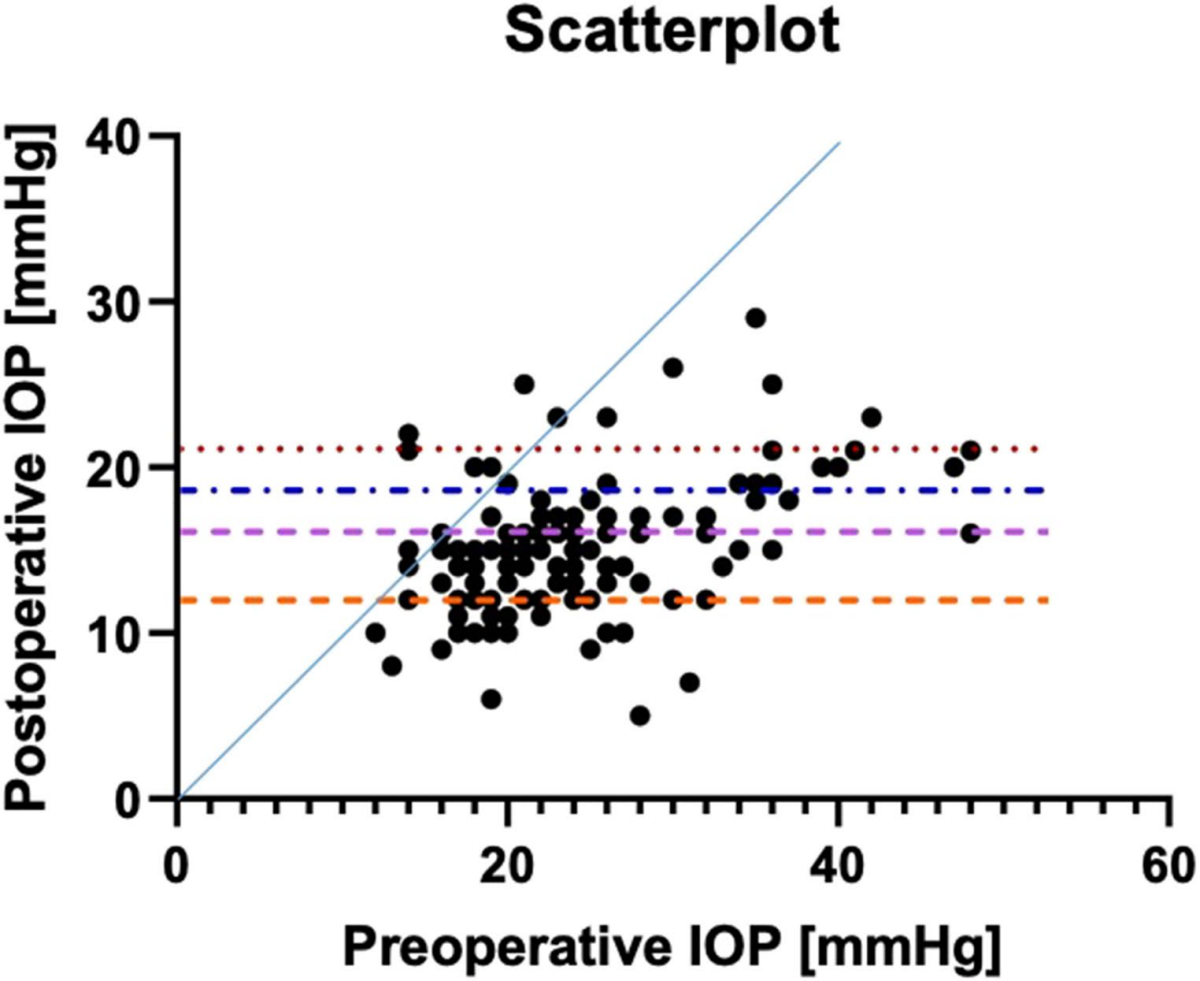
Scatterplot of preoperative versus postoperative intraocular pressure (IOP) values at the last follow-up of each patient. Dots represent eyes that underwent canaloplasty. The dotted lines mark the 21 mmHg, 18 mmHg, 15 mmHg, and 12 mmHg thresholds

The preoperative best-corrected visual acuity was 0.2 ± 0.2 logMAR and did not significantly change throughout the follow-up period (12-month follow-up: 0.2 ± 0.3 logMAR, *p* = 0.117) (Fig. 2).

### Identification of critical thresholds

**Fig. 3:**
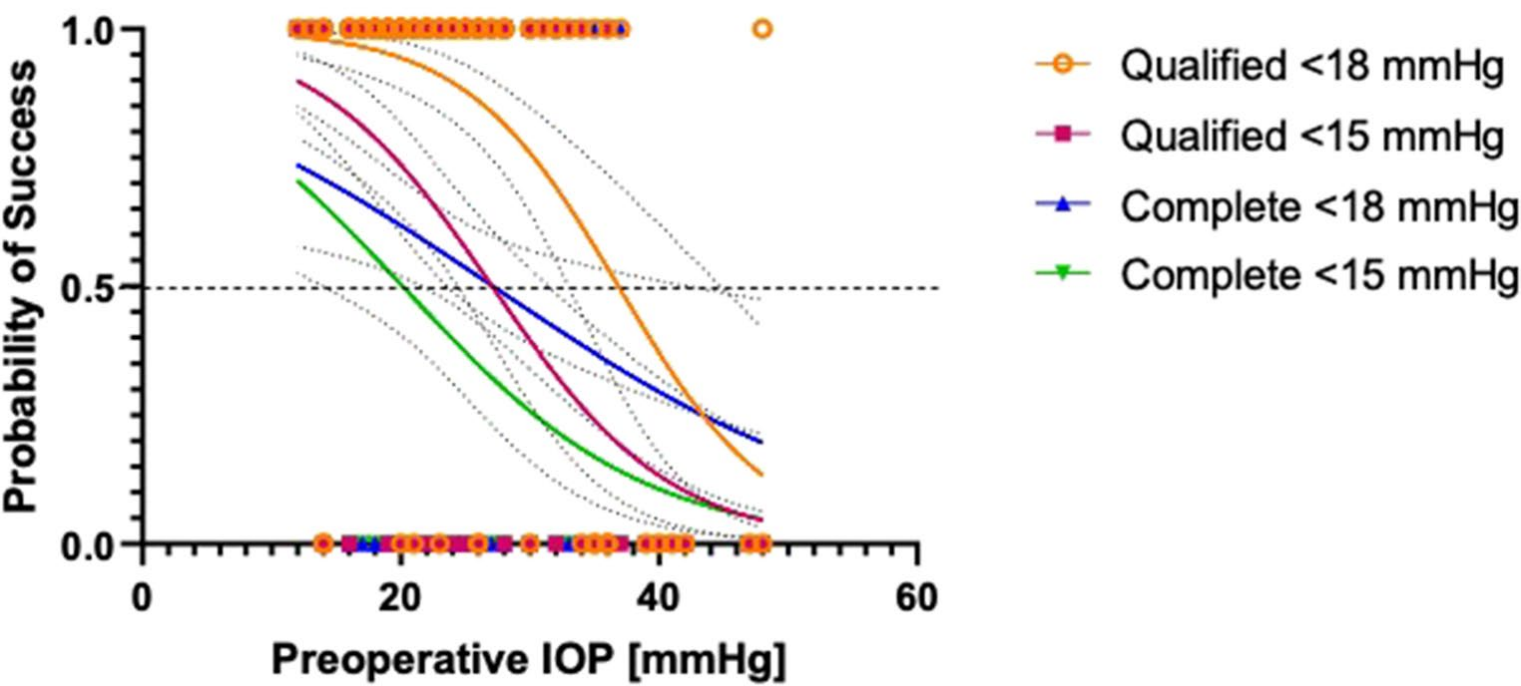
Regression curves illustrating the predicted probability of surgical success based on preoperative intraocular pressure (IOP). Four curves are shown corresponding to the following success definitions: complete success (IOP ≤ 18 mmHg and IOP ≤ 15 mmHg) and qualified success (IOP ≤ 18 mmHg and IOP ≤ 15 mmHg). The x-axis represents the preoperative IOP, while the y-axis (ranging from 0 to 1) indicates the predicted probability of success (1: surgical success)

**Fig. 4:**
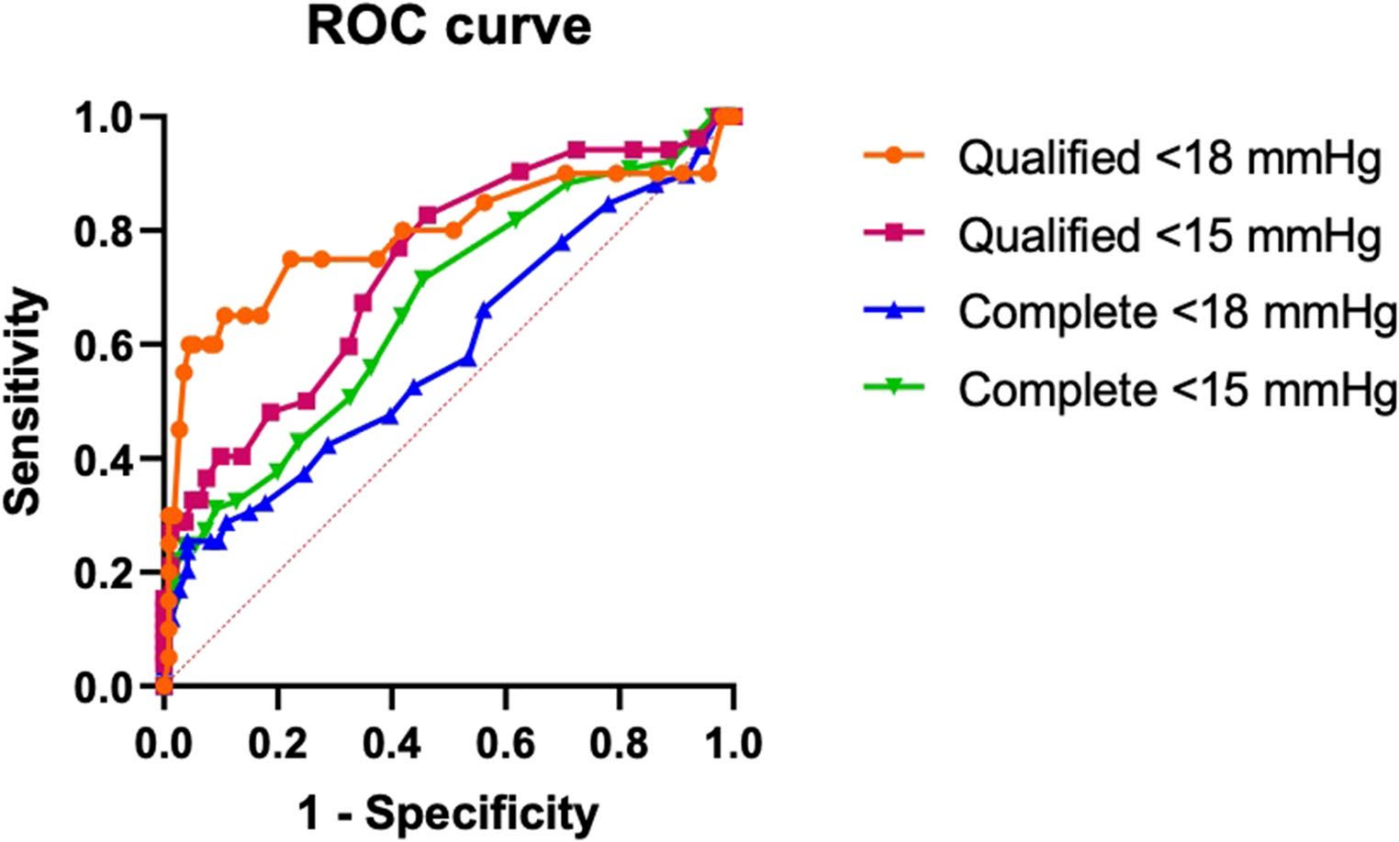
Receiver operating characteristic (ROC) curves for predicting surgical success based on preoperative intraoperative pressure (IOP). The analyses include four surgical success definitions: complete success (without eye drops, for IOP ≤ 18 mmHg and IOP ≤ 15 mmHg) and qualified success (with or without eye drops, for IOP ≤ 18 mmHg and IOP ≤ 15 mmHg)

Logistic regression analysis was conducted to identify preoperative IOP thresholds (X at 50% values) associated with surgical failure for the different success definitions. Specifically, patients with a preoperative IOP ≥ 27.1 mmHg had a higher risk of failure for complete success at ≤ 18 mmHg (Table 2). Similarly, an IOP threshold of 20.1 mmHg was identified for complete success at ≤ 15 mmHg. For qualified success, the identified thresholds were 27.1 mmHg for target pressures ≤ 18 mmHg and 20.1 mmHg for ≤ 15 mmHg. These results are detailed in Table 2; Figs. 3 and 4. No significant associations were found between preoperative IOP and complete or qualified success rates for IOP ≤ 21 mmHg or IOP ≤ 12 mmHg. Additionally, logistic regression analysis showed no statistically significant association between the number of preoperative IOP-lowering eye drops and the risk of surgical failure.

**Table 2:** Logistic regression analysis of preoperative intraocular pressure (IOP) threshold values for surgical success, including preoperative IOP and 95% confidence intervals (CI), p-values, and area under the receiver operating characteristic (ROC) curve

Success definition	X at 50% (Preoperative Threshold IOP)	95% CI	*p*-value	Area under the ROC curve
Qualified ≤ 18 mmHg	**36.9 mmHg**	32.92–44.21	*p* < 0.001	0.7893
Qualified ≤ 15 mmHg	**27.1 mmHg**	24.40–31.05	*p* < 0.001	0.7365
Complete ≤ 18 mmHg	**27.1 mmHg**	21.72–40.26	*p* = 0.0109	0.5890
Complete ≤ 15 mmHg	**20.1 mmHg**	14.25–23.55	*p* = 0.0011	0.6656

#### Kaplan-Meier survival analysis

**Fig. 5:**
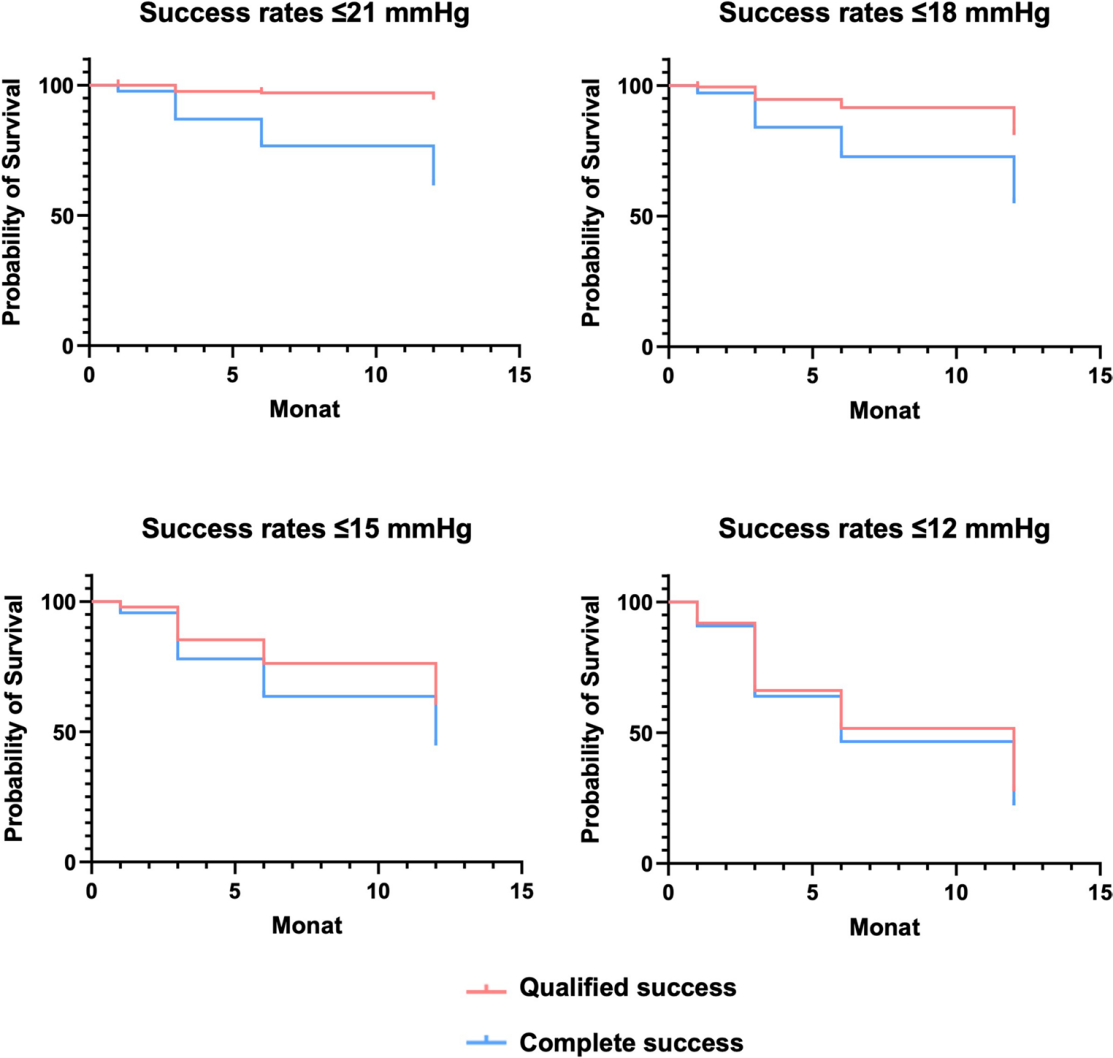
Kaplan Meier curves for complete and qualified success rates (≤ 21 mmHg, ≤ 18 mmHg, ≤ 15, and ≤ 12 mmHg) during 12 months following canaloplasty

Kaplan–Meier curves illustrate the cumulative probability of maintaining surgical success over 12 months for different intraocular pressure (IOP) thresholds (Fig. 5). At the 12-month follow-up, complete success rates were 61.5% for IOP ≤ 21 mmHg, 55.0% for IOP ≤ 18 mmHg, 44.7% for IOP ≤ 15 mmHg, and 22.3% for ≤ 12 mmHg (Fig. 5). Qualified success rates were 94.4% for IOP ≤ 21 mmHg, 81.1% for IOP ≤ 18 mmHg, 60.4% for IOP ≤ 15 mmHg, and 27.6% for ≤ 12 mmHg.

#### Complications and additional interventions

No significant intraoperative complications were observed. Among the 166 patients undergoing canaloplasty, *n* = 6 (3.6%) experienced numerical hypotony; however, no patient developed clinical hypotony requiring intervention. A postoperative, self-limiting hyphema was observed in *n* = 49 (29.5%) patients, and corneal erosion occurred in *n* = 2 (1.2%) patients. Further glaucoma interventions were necessary due to uncontrolled IOP in *n* = 13 (7.8%) patients; of these, *n* = 9 (5.4%) underwent a 360° trabeculotomy, and *n* = 4 (2.4%) required reoperations, including *n* = 3 (1.8%) trabeculectomies and *n* = 1 (0.6%) glaucoma drainage implant (all at the 12-month follow-up). All patients who required a subsequent IOP-lowering surgery were excluded from the study from the time of that procedure onward.

## Discussion

This study aimed to establish preoperative thresholds and to assess the predictive value of IOP and the number of IOP-lowering eye drops on surgical success of canaloplasty. Over a 12-month follow-up period, mean IOP decreased from 24.2 ± 7.8 mmHg preoperatively to 14.8 ± 3.7 mmHg at 12 months (relative IOP reduction of 34.0%), while the number of IOP-lowering eye drops decreased from 2.3 ± 1.1 to 0.6 ± 1.0. Logistic regression analysis further identified preoperative IOP thresholds above which the risk of surgical failure increased significantly for both complete and qualified success definitions. In contrast, no significant association was found between the number of preoperative IOP-lowering eye drops and surgical success.

The IOP reduction observed in our study is consistent with previous reports on canaloplasty. Matlach et al. demonstrated a significant decrease in IOP from 23.7 ± 5.1 mmHg to 13.8 ± 2.7 mmHg at 12 months in a cohort of 23 patients, which is comparable to the reduction observed in our study (from 24.2 ± 7.8 mmHg to 14.8 ± 3.7 mmHg) [9]. Similarly, Bull et al. reported an IOP reduction from 23.0 ± 4.3 mmHg to 15.5 ± 3.5 mmHg 12 months after canaloplasty in 73 glaucoma patients [11].

In addition, our study found a significant decrease in the number of IOP-lowering medications, with the mean number of eye drops decreasing from 2.3 ± 1.1 to 0.6 ± 1.0 at the 12-month follow-up. This result is in agreement with previous studies, which have reported reductions from 2.6 ± 1.6 to 0.8 ± 1.2 eye drops [9] and from 1.9 ± 0.7 to 0.6 ± 0.9 eye drops [11].

The qualified success rates for IOP ≤ 21 mmHg, ≤ 18 mmHg, and ≤ 15 mmHg at the 12-month follow-up were 94.4%, 81.1%, and 60.4%, respectively, in our study. These outcomes are comparable to those reported by Bull et al., who observed success rates of 95.9%, 76.7%, and 52.1% 12 months after canaloplasty [11]. Similarly, Grieshaber et al. reported qualified success rates of 86.9% for IOP ≤ 21 mmHg, 80.6% for IOP ≤ 18 mmHg, and 66.3% for IOP ≤ 16 mmHg 12 months post-canaloplasty [12].

Our logistic regression analysis identified preoperative IOP thresholds of 36.9 mmHg, 27.1 mmHg, 27.1 mmHg, and 20.1 mmHg for the different definitions of success (complete and qualified success for IOP targets ≤ 18 and ≤ 15 mmHg, respectively). These thresholds suggest that patients with preoperative IOPs below these values are more likely to achieve the corresponding target IOPs postoperatively.

The concept of calculating baseline IOP thresholds to predict treatment outcomes in glaucoma interventions based on logistic regression has been suggested before. Hodge et al., using a model based on logistic regression, demonstrated that preoperative IOP is a significant predictor of selective laser trabeculoplasty (SLT) success at one year, whereas factors such as age, sex, and family history of glaucoma did not show a significant impact [13]. Specifically, Hodge et al. demonstrated that lower baseline IOP predicts lower post-treatment IOP, and that patients with higher baseline IOP benefitted more from the SLT in terms of total IOP reduction [13]. These findings have been corroborated by other studies, which similarly indicate greater IOP lowering effects of SLT and prostaglandin analogue eye drops in patients with higher preoperative IOP [14].

To our knowledge, no previous study has applied logistic regression to determine preoperative IOP thresholds predictive of canaloplasty success. Identifying robust preoperative predictive factors in the management of glaucoma could enable clinicians to tailor therapeutic strategies to individual patient profiles. A predictive algorithm would facilitate the optimal selection of candidates for canaloplasty and forecasting surgical outcomes after glaucoma surgery [15, 16].

In our study, we built on this foundation by employing logistic regression to determine specific baseline IOP thresholds for predicting the success of canaloplasty. Our approach reinforces the prognostic value of preoperative IOP, as evidenced in prior work, and refines it by providing suggestive threshold values that can enhance preoperative evaluation, candidate selection, and ultimately, individualized treatment planning for glaucoma patients.

Our analysis did not reveal a significant association between the number of preoperative IOP-lowering eye drops and the risk of surgical failure. This suggests that the absolute level of IOP, rather than the intensity of pharmacological therapy, is a more reliable predictor of surgical outcomes. Within the context of logistic regression, we identified significant preoperative IOP values for predicting both complete and qualified success at ≤ 18 mmHg and ≤ 15 mmHg. In contrast, the analysis did not yield statistically significant results for complete or qualified success rates for IOP ≤ 21 mmHg and ≤ 12 mmHg. One possible explanation for this finding is that the ≤ 21 mmHg target may encompass a broad range of postoperative outcomes that dilute the predictive power of baseline IOP. Since many patients achieve IOP reductions well below this level, an IOP ≤ 21 mmHg may not serve as a strong enough discriminator between success and failure. Conversely, the ≤ 12 mmHg target may be too restrictive, with few patients reaching such low postoperative IOP levels after canaloplasty, leading to a reduced sample size and, consequently, limited statistical power to detect a significant effect. We agree with previous authors [17] that the primary limitation of canaloplasty stems from its reliance on the resistance of the distal outflow system and episcleral venous pressure. Consequently, achieving target IOPs below 12 mmHg is unlikely with this procedure. These results suggest that mid-range IOP targets, such as ≤ 18 mmHg and ≤ 15 mmHg, may be more clinically relevant for defining surgical success in canaloplasty, as they hypothetically better capture the range of IOP reductions typically achieved. Further studies with larger cohorts may help refine these thresholds and enhance their applicability in preoperative decision-making.

This study has several limitations. First, the follow-up period of our study is limited to 12 months after canaloplasty. Longer follow-up studies are necessary to determine whether these preoperative thresholds remain predictive over extended periods and to assess the sustainability of the IOP-lowering effect. Second, although our sample size of 166 patients is substantial, larger cohorts would further enhance the statistical power of our analyses, particularly in refining predictive models and validating the identified IOP thresholds. Also, more extensive datasets would allow for more robust subgroup analyses, potentially identifying additional prognostic factors.

Additionally, the retrospective design of our study introduces inherent limitations, including potential selection bias. Lastly, we primarily focused on IOP-related success criteria; incorporating functional outcomes such as visual field progression or patient-reported quality-of-life measures could offer a more holistic assessment of canaloplasty’s effectiveness. Future studies investigating these aspects could further validate the preoperative IOP thresholds and strengthen their clinical relevance.

## Conclusion

This study highlights the predictive value of preoperative IOP thresholds in determining the success of canaloplasty, offering valuable insights for optimizing patient selection. Our findings suggest that in patients with preoperative IOPs of ≤ 36.9 mmHg or ≤ 27.1 mmHg, canaloplasty is likely to achieve target pressures of ≤ 18 mmHg, with or without the need for IOP-lowering eye drops, respectively. These results reinforce the role of baseline IOP as a key determinant of surgical success and emphasize the need for individualized treatment planning in glaucoma management.
